# Circulating tumor cell detection and single‐cell analysis using an integrated workflow based on ChimeraX^®^‐i120 Platform: A prospective study

**DOI:** 10.1002/1878-0261.12876

**Published:** 2020-12-25

**Authors:** Peng‐Xiang Wang, Yun‐Fan Sun, Wei‐Xiang Jin, Jian‐Wen Cheng, Hai‐Xiang Peng, Yang Xu, Kai‐Qian Zhou, Li‐Meng Chen, Kai Huang, Sui‐Yi Wu, Bo Hu, Ze‐Fan Zhang, Wei Guo, Ya Cao, Jian Zhou, Jia Fan, Xin‐Rong Yang

**Affiliations:** ^1^ Department of Liver Surgery & Transplantation Liver Cancer Institute Zhongshan Hospital Fudan University Shanghai China; ^2^ Key Laboratory of Carcinogenesis and Cancer Invasion Ministry of Education Shanghai China; ^3^ Shanghai Epione Medlab China; ^4^ Department of Laboratory Medicine Zhongshan Hospital Fudan University Shanghai China; ^5^ Key Laboratory of Carcinogenesis and Cancer Invasion Cancer Research Institute Central South University Ministry of Education Changsha China; ^6^ Institutes of Biomedical Sciences Fudan University Shanghai China

**Keywords:** circulating tumor cell, enumeration, integrated platform, liquid biopsy, machine learning‐based image recognition, single‐cell sequencing

## Abstract

Circulating tumor cell (CTC) analysis holds great potential to be a noninvasive solution for clinical cancer management. A complete workflow that combined CTC detection and single‐cell molecular analysis is required. We developed the ChimeraX^®^‐i120 platform to facilitate negative enrichment, immunofluorescent labeling, and machine learning‐based identification of CTCs. Analytical performances were evaluated, and a total of 477 participants were enrolled to validate the clinical feasibility of ChimeraX^®^‐i120 CTC detection. We analyzed copy number alteration profiles of isolated single cells. The ChimeraX^®^‐i120 platform had high sensitivity, accuracy, and reproducibility for CTC detection. In clinical samples, an average value of > 60% CTC‐positive rate was found for five cancer types (i.e., liver, biliary duct, breast, colorectal, and lung), while CTCs were rarely identified in blood from healthy donors. In hepatocellular carcinoma patients treated with curative resection, CTC status was significantly associated with tumor characteristics, prognosis, and treatment response (all *P* < 0.05). Single‐cell sequencing analysis revealed that heterogeneous genomic alteration patterns resided in different cells, patients, and cancers. Our results suggest that the use of this ChimeraX^®^‐i120 platform and the integrated workflow has validity as a tool for CTC detection and downstream genomic profiling in the clinical setting.

## Graphical abstract

We developed an integrated workflow for circulating tumor cell (CTC) detection and downstream single‐cell analysis based on a novel ChimeraX^®^‐i120 platform. The platform facilitates negative enrichment, immunofluorescent labeling, and machine learning‐based identification of CTCs. The CTC captured by the platform is also compatible for single‐cell molecular analysis. In this study, potential utility of our workflow was validated in clinical setting.

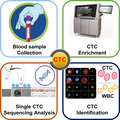

**Abbreviations**
ADABOOST: AdaBoost classification trees
AFP: alpha‐fetoprotein
AUC: areas under the curve
BC: breast cancer
BCLC: barcelona clinic liver cancer
BHL: benign hepatic lesion
CCD: charge‐coupled device
CHB: chronic hepatitis B
CK: cytokeratin
CNA: copy number alteration
CNLC: Chinese staging for liver cancer
CRC: colorectal cancer
CTC: circulating tumor cell
CTM: circulating tumor microemboli
CV: coefficient of variation
DAPI: 4’,6‐diamidine‐2’‐phenylindole dihydrochloride
EpCAM: epithelial cell adhesion molecule
FPR: false‐positive rate
GBM: stochastic gradient boosting
HCC: hepatocellular carcinoma
HD: healthy donor
ICC: intrahepatic cholangiocarcinoma
LC: liver cirrhosis
LCA: lung cancer
LOD: limit of detection
PBS: phosphate‐buffered saline
PCR: polymerase chain reaction
RF: random forest
ROC: receiver operating characteristic
SVM: support vector machines
TCGA: The Cancer Genome Atlas
TPR: true‐positive rate
TTR: time to recurrence
WBC: white blood cell
WGA: whole‐genome amplification
WGS: whole‐genome sequencing
XGB: extreme gradient boosting

## 1 Introduction

Cancer metastasis is the main cause of cancer‐related deaths worldwide [1]. Circulating tumor cells (CTCs) are rare cancer cells that circulate in the bloodstream after they are shed from tumors. CTCs are regarded as ‘seeds’ that initiate cancer progression and metastasis [2, 3]. Characterizing these rare cells from blood represents a potential surrogate for tumor biopsy and may provide crucial information related to cancer progression, prognostication, and therapeutic response [4, 5]. Furthermore, moving beyond the simple enumeration of CTCs toward more sophisticated single‐cell molecular analyses can fully realize their potential as biomarkers used to understand underlying mechanisms of cancer metastasis and develop new therapeutic strategies [6, 7, 8, 9].

Due to the scarcity of CTCs and the contamination of other blood cells, CTCs are generally required to be enriched from the patient’s blood sample before further analysis. CTC enrichment methods are classified based on whether they use the physical or biological properties of the target cells [10]. Many microfluidic platforms have been developed in the past few years as a representative form of physical properties‐based CTC sorting, which allows for rapid and precise CTC capture [11, 12]. Currently, label‐dependent positive separation remains a major method used in CTC enrichment. CellSearch system, the most frequently used and the only US FDA‐approved semi‐automated CTC detection device, enriched CTCs based on the expression of the surface marker epithelial cell adhesion molecule (EpCAM) [13, 14]. However, surface antigen‐dependent CTC capture methods may cause great loss of CTCs due to low or negative expression of tumor epithelial cell markers [15, 16]. These traditional platforms are also generally incompatible with direct downstream single‐cell molecular analyses, which further limit their clinical utility for comprehensive CTC‐based analysis [13, 17].

Molecular profiling of CTCs is also extremely challenging due to technical difficulties [18]. Technologies for CTC capture and downstream single‐cell molecular profiling are generally designed for scientific research. They require multiple batch‐process steps and have relatively limited throughput. A standardized workflow that facilities integrated CTC enrichment, enumeration, and single‐cell analysis is needed.

**Fig. 1:**
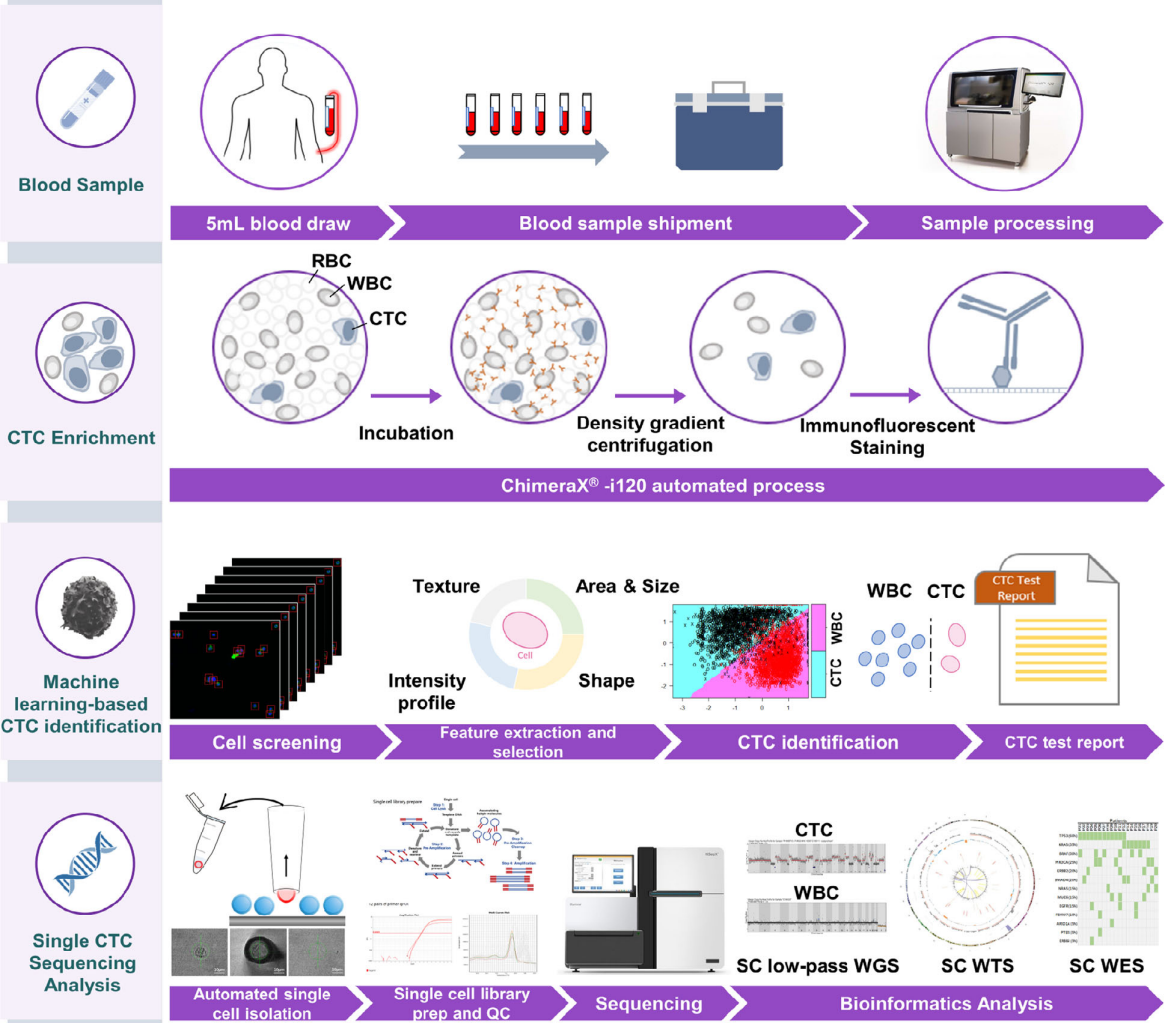
Sample preparation, CTC enrichment, identification, single CTC sequencing analysis workflow.

**Fig. 2:**
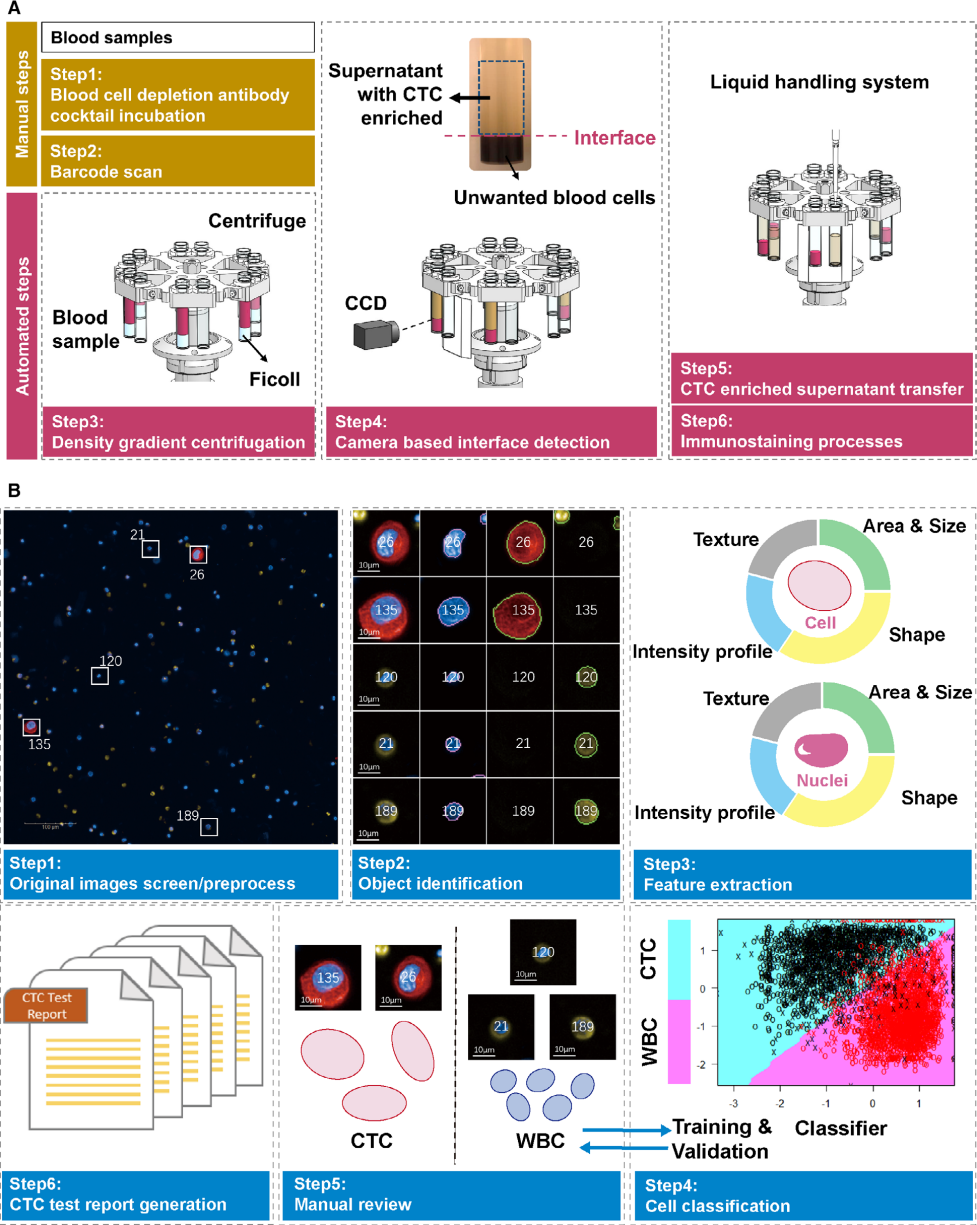
Detection and identification of CTCs using ChimeraX^®^‐i120 platform. (A) Automatic CTC enrichment and staining process. (B) Machine learning‐based CTC identification. Scale bar, 10 μm.

Since populations of CTCs are highly heterogeneous, negative enrichment might be a more reasonable strategy for CTC detection [16, 19]. In this approach, nontargeted blood cells (i.e., erythrocytes, leukocyte, and platelets) are eluted, and targeted CTCs are captured. Using this strategy, intact tumor cells can be isolated, characterized, and even cultured, which is an essential prerequisite for sophisticated downstream analyses [20]. Here, an automatic platform, ChimeraX^®^‐i120, was developed to address the limitations of label‐dependent CTC enrichment (Fig. 1). Our integrated approach for CTC detection included negative enrichment, immunofluorescent labeling, and machine learning‐based CTC identification (Fig. 2A,B). The results of CTC enumeration demonstrated the potential utility of this platform for different cancer types. A complete workflow for downstream single‐cell micromanipulation and genomic characterization of the isolated CTC was established and tested for its feasibility. Our integrated standardized workflow represents a promising solution for precise CTC enumeration and molecular profiling at the single‐cell level in the clinical setting.

## 2 Materials and methods

### 2.1 Participants enrollment and blood sample collection

A total of 477 participants were prospectively enrolled in the study (Zhongshan Hospital) from October 2017 to September 2019. The study population included 281 patients with different types of cancer, 71 patients with chronic hepatitis B infection/liver cirrhosis (CHB/LC, *n* = 31), and benign hepatic lesion (BHL, *n* = 40), and 125 healthy donors (HDs). The enrolled patients with cancer, including hepatocellular carcinoma (HCC, *n* = 145), intrahepatic cholangiocarcinoma (ICC, *n* = 38), breast cancer (BC, *n* = 44), colorectal cancer (CRC, *n* = 39), and lung cancer (LCA, *n* = 15), accepted CTC testing before any palliative or curative treatments were given. The diagnoses of the different diseases were based on histological or typical imaging analysis results. Tumor stage was defined based on 8th edition of American Joint Committee on Cancer guidelines [21]. HDs without evidence of a history of malignancy or systemic diseases were enrolled for the analytical assay and clinical performance validation of the CTC test.

The inclusion criteria for the patients with HCC were as follows: (1) HCC confirmed by histological examination; (2) no history of other malignancies; (3) no previous anticancer treatment; (4) accepted curative resection, defined as all macroscopic lesions removed using partial liver resection [22]; and (5) absence of extrahepatic metastasis. HCC tumor stage was classified according to Barcelona Clinic Liver Cancer (BCLC) staging system [23] and Chinese staging for Liver Cancer (CNLC) guidelines [24]. The Edmondson grading system was used to define HCC differentiation [25].

The venipuncture for CTC detection was performed following a standard procedure that the first 2 mL of blood was discarded to avoid possible contamination of epithelial skin cells. Then, peripheral blood (5 mL samples) was collected into Vacutainer K_2_EDTA tubes (BD Bioscience, USA) and processed within 12 h using the ChimeraX^®^‐i120 platform.

The study was approved by the Institutional Ethics Committee of Zhongshan Hospital, Fudan University, conformed to the ethical guidelines of the 1975 Declaration of Helsinki. Written informed consent was obtained from every participant.

### 2.2 Follow‐up and tumor progression for patients with HCC

For patients with HCC, follow‐ups occurred every 2–3 months during the first year after surgery and every 3–4 months thereafter; follow‐ups ended in July 2020. Patients were monitored by serum alpha‐fetoprotein (AFP) levels, abdominal ultrasonography, chest radiography, and abdominal computed tomography or magnetic resonance imaging regularly based on postoperative time. HCC recurrence was diagnosed based on cytologic/histologic evidence or noninvasive diagnostic criteria according to the Guidelines for Diagnosis and Treatment of Primary Liver Cancer in China (2017 edition) [24]. Time to recurrence (TTR) was defined as the interval between resection and diagnosis of any type of recurrence.

### 2.3 CTC enrichment and labeling using the ChimeraX^®^‐i120 platform

Each blood sample was first incubated at room temperature for 10 min with 60 μL·mL^−1^ of a bifunctional antibody cocktail containing tetrameric antibody complexes, to specifically conjugate and deplete unwanted leukocyte subsets and red blood cells (Genovo, GEN‐304777, San Diego, CA, USA). The sample was then diluted with equal volumes of phosphate‐buffered saline (PBS) and carefully layered on top of 3.5 mL Ficoll–Paque PLUS (*d* = 1.077 g·mL^−1^) (GE Healthcare, Pittsburgh, PA, USA). The sample barcode was then scanned, and the sample was transferred into the ChimeraX^®^‐i120 platform. Samples in the system were processed using density gradient centrifugation (Video S1). Each sample was separated into four visible layers (unwanted blood cells, Ficoll, buffy coat, and plasma) after the centrifugation was completed. The centrifuge buckets held the sample tubes on the top so that a charge‐coupled device (CCD) camera could take snapshots of the samples, recognizing the interface between the blood cell layer and Ficoll, and calculate the volume of supernatant. To minimize cell loss, the liquid handling system then transferred all the supernatant (containing Ficoll, buffy coat, and plasma) to a new tube and proceeded with the immunostaining steps (Fig. 2A).

During the immunofluorescence staining steps, each sample was automatically fixed, washed, and stained with a cocktail of 4’,6‐diamidine‐2’‐phenylindole dihydrochloride (DAPI, Genovo, GEN‐D1306), Alexa Fluor 647‐labeled mouse anti‐human cytokeratin 19 (CK19, Genovo, GEN‐628502)/pan‐cytokeratin (pan‐CK, Genovo, GEN‐628601)/EpCAM (Genovo, GEN‐511001), and Alexa Fluor 555‐labeled mouse anti‐human CD45 antibodies (Genovo, GEN‐304056) (all 2.5 μg·mL^−1^). Permeabilization and blocking reagents were added in the washing buffer and staining buffer, to reduce steps required and consequent cell loss. Stained samples were output to 96‐well plates and scanned using a CellInsight CX5 High‐Content Screening Platform (Thermo Fisher Scientific, Waltham, MA, USA). Detected CTCs were defined as nucleated (DAPI^+^) intact cells with positive pan‐CK/CK19/EpCAM and negative for CD45.

### 2.4 Image analysis and machine learning‐based CTC identification

Image analysis for picture segmentation and feature extraction was performed using a customized pipeline built in CellProfiler (Broad Institute, USA). The original fluorescent images were preprocessed to correct uneven illumination and remove noise. The OTSU thresholding method was then applied to distinguish foreground signals from background signals and to identify outlines of objects [26]. More than 700 cellular features (e.g., marker expression and cell morphology) were recognized, extracted, and quantified. A cytological profile was then created for each cell. Filters based on cellular feature measurements were used to remove debris and to only include cells in the identified objects (Fig. 2B).

Based on these cellular features, five classifiers including stochastic gradient boosting (GBM), AdaBoost classification trees (ADABOOST), support vector machines (SVM), random forest (RF), and extreme gradient boosting (XGB) were implemented and compared to rapidly classify cells as CTC candidates, or not [27]. All code for the machine learning algorithm was written in R 3.1.0 (R Foundation for Statistical Computing, Austria). All the classifiers were developed using caret, a machine learning library in R (http://topepo.github.io/caret/index.html). An in‐house manually annotated CTC test dataset was randomly split into training (75% data, with 10‐fold cross‐validation) and testing (25% data) sets for classifier development and assessment (Fig. S1). The overall performance of classifiers was evaluated using receiver operating characteristic (ROC) analyses, accuracy, precision, recall, F1 score, true‐positive rate (TPR), and false‐positive rate (FPR) [27]. The identities of the cells identified by the algorithm were confirmed by two trained pathologists (Video S2).

### 2.5 Cell lines and culture

Four human cancer cell lines with different EpCAM expression levels (i.e., SkBr3, HT‐29, HepG2, and T24 cell lines) were purchased from the Typical China Academy Culture Collection Commission Cell Library (Shanghai, China). Cell lines were maintained in an incubator (37 °C and 5% CO_2_). Passages were performed when cells reached 70–80% confluency in a T‐25 flask. Using the image analysis pipeline, the relative EpCAM expression levels in four cancer cell lines were quantified based on average fluorescence intensities after staining with EpCAM‐AF647 antibodies.

### 2.6 Analytical performance validation of CTC detection

Accuracy, limit of detection (LOD), precision, specificity, and anti‐interference capability of the ChimeraX^®^‐i120 platform in CTC detection were assessed (Table 1) [28]. In the assay, SkBr3 cells were harvested with 0.25% trypsin‐EDTA (Gibco, Grand Island, NY, USA), spiked into the blood samples from HDs, and processed using the ChimeraX^®^‐i120 platform. Recovery rate (%) = (number of cells recovered/number of cells spiked) ×100.

**Table 1:** Description of characteristics in analytical validation of ChimeraX^®^‐i120 platform

Characteristics	Samples	Assessment
Accuracy	Spiked peripheral blood from healthy donors with a range of cells (0–250), repeat for 5 days	Average recovery rate linearity of detected cell numbers
Limit of detection (LOD)	Spiked peripheral blood from healthy donors with a range of cells (0–5), repeat for 10 times	Lowest measurable CTC count per 5 mL blood
		
Precision (reproducibility)	Spiked peripheral blood from healthy donors with 20 cells processed by 2 operators in 3 days, repeat for 3 times	Intra‐assay and interassay variability calculated by coefficient of variation (%CV)
		
Specificity	Peripheral blood from healthy donors, repeat 10 days.	False‐positive detection rate
Anti‐interference capability	Spiked peripheral blood (20 cells) from healthy donors with or without endogenous interfering reagents (bilirubin,triglycerides, hemoglobin) at a high level, repeat for 3 times	Difference in recovery rates between the interfering group and the control group

### 2.7 Detection of CTCs using the CellSearch system

Positive enrichment and enumeration of EpCAM^+^ CTCs in preclinical spike‐in experiments and clinical samples were performed using a CellSearch system (Menarini Silicon Biosystems, Huntington Valley, PA, USA), as previously described [14]. The CTC enumeration results were presented as the numbers of cells per 7.5 mL blood.

### 2.8 Single CTC micromanipulation, library preparation, and sequencing

CTCs enriched by the ChimeraX^®^‐i120 platform were transferred into polymerase chain reaction (PCR) tubes containing cell lysis buffer using an automated micromanipulation platform, CellCelector™ (ALS, Jena, Germany). Single‐cell whole‐genome amplification (WGA) and the sequencing library were prepared using the SMARTer^®^ PicoPLEX^®^ Gold Single‐Cell DNA‐Seq Kit (Takara Biosystems, Tokyo, Japan), according to the manufacturer’s instructions. The libraries were quantified using Qubit dsDNA HS Assay Kits with a Qubit 2.0 Fluorometer (Thermo Fisher Scientific). Fragment analysis was performed using Agilent High Sensitivity DNA Kits and an Agilent Bioanalyzer 2100 (Agilent Technologies, Santa Clara, CA, USA). Quantitative PCR (qPCR) was performed on 12 randomly selected loci on different chromosomes to evaluate the genomic integrity of the amplification products. The library with at least eight out of 12 loci amplified at an expected melting temperature (Tm) and cycle threshold (Ct) number < 30 was subjected to sequencing.

For tissue samples, DNA was extracted using AllPrep DNA/RNA Mini Kits (Qiagen, Duesseldorf, Germany). DNA libraries were prepared using TruSeq DNA HT Sample Prep Kits (Illumina, San Diego, CA, USA) with 100 ng DNA added per library preparation. The library was sequenced using an Illumina HiSeq X Ten System (read lengths: 2 × 150 bp, average depth: 0.5–2.0×).

### 2.9 Bioinformatic analysis

Raw data were first mapped to the UCSC human reference genome hg19 using BWA 0.7.17. After alignment, samtools 1.7 software was used to sort the alignment files to sorted.bam files. These files were marked duplicate by Picard 2.18.0. The output reads data were then analyzed using Ginkgo (http://qb.cshl.edu/ginkgo), an open‐source platform suitable for analysis of low‐depth sequencing coverage of single‐cell copy number alteration (CNA). Quality metrics data, including index of dispersion, Lorenz curves, and histograms of read count distributions, were calculated as a part of the Ginkgo analysis pipeline. Putative cancer driver genes among the CNA regions were analyzed based on a recent study on The Cancer Genome Atlas (TCGA) database [29].

### 2.10 Statistical analysis

All results were presented as mean ± standard deviation values. Between‐group comparisons for individual variables were performed using Student’s *t* tests, Wilcoxon signed‐rank tests, or Mann–Whitney *U* tests, where appropriate. Categorical data were analyzed using chi‐squared tests or Fisher’s exact tests. The diagnostic value of CTCs was estimated by ROC curve analysis. Cumulative recurrence rates were estimated by the Kaplan–Meier analysis; differences between groups were assessed using log‐rank tests. Two‐sided values of *P* < 0.05 were considered statistically significant. spss 24.0 for windows (IBM, Armonk, NY, USA) was used for the statistical analysis.

## 3 Results

### 3.1 Analytical evaluation of ChimeraX^®^‐i120 platform by spike‐in experiments

**Fig. 3:**
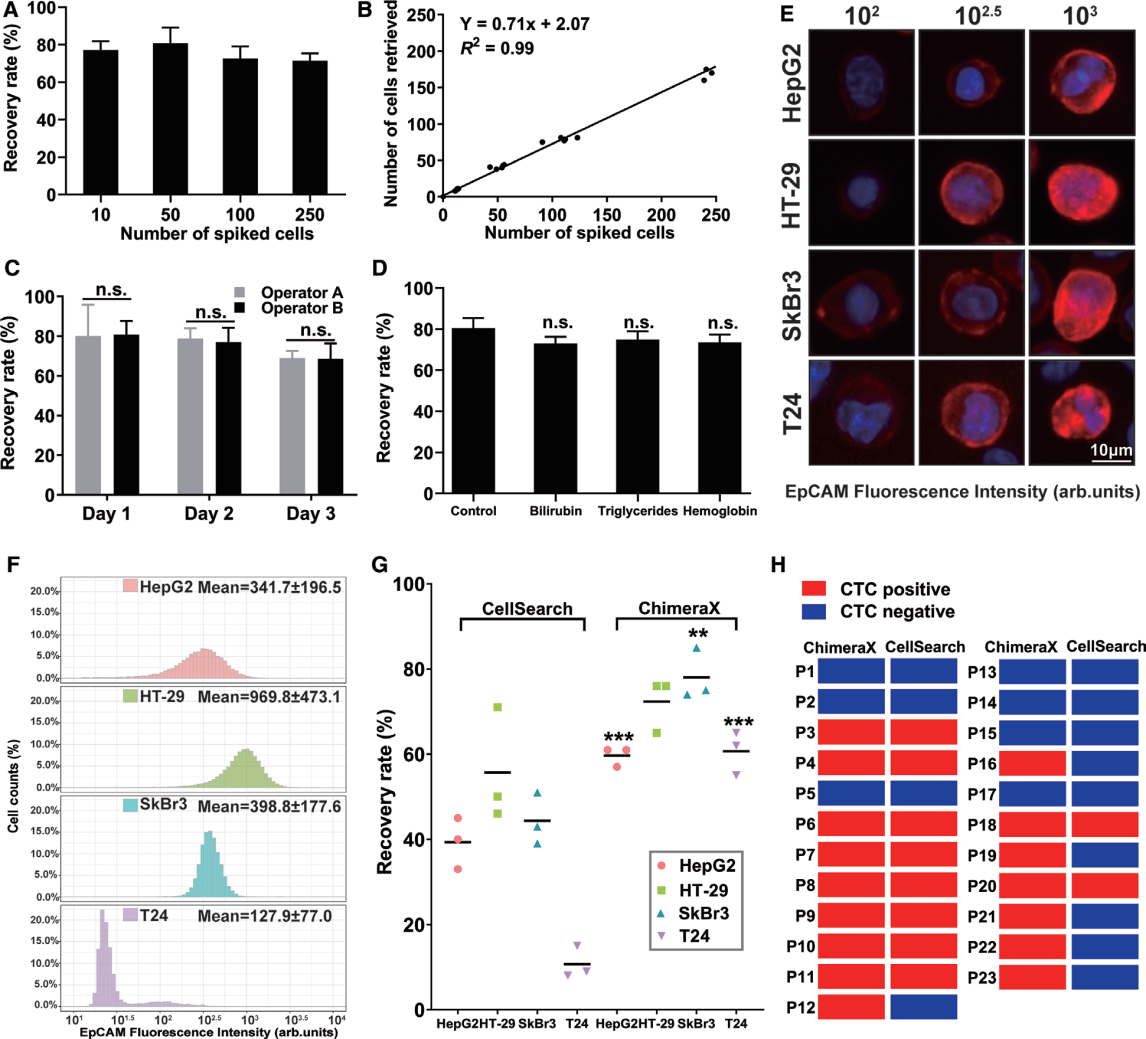
Construction and evaluation of ChimeraX^®^‐i120 CTC detection platform. (A) Recovery efficiency of 0–250 spiked SkBr3 cells in 5mL blood by ChimeraX^®^‐i120 platform (*n* = 5). (B) Linear regression analysis. (C) Precision analysis of ChimeraX^®^‐i120 platform performed in multiple days (n = 3) by two operators (*n* = 2) using paired blood samples, repeated for three times. (D) Anti‐interference experiment by adding endogenous interfering reagents to the blood, repeated for three times. (E) Typical images of four cancer cell lines when stained by EpCAM‐AF647 antibody. (F) Relative EpCAM expression level of SkBr3, HT‐29, HepG2, and T24 cell lines measured by average fluorescence intensity. (G) Comparison of recovery rate between ChimeraX^®^‐i120 platform and CellSearch system of spiked four cancer cell lines (T24, HepG2, SkBr3, HT‐29). (H) Comparison of ChimeraX^®^‐i120 platform with CellSearch system in paired clinical samples from cancer patients (*n* = 23). Each error bar represents the standard deviation (SD). Statistical analysis was performed using the unpaired Student’s *t*‐test. Significance is indicated by ^**^ *P* < 0.010 and ^***^ *P* < 0.001. Scale bar, 10 μm.

Analytical performance of the ChimeraX^®^‐i120 platform for CTC detection was evaluated using a series of spike‐in experiments at varying cell concentrations. An average recovery rate of 75.6% (range, 71.3–83.2%) was obtained for 10, 50, 100, and 250 spiked cells (Fig. 3A). Linear regression analysis revealed excellent assay linearity (*R* ^2^ = 0.99, Fig. 3B). A single tumor cell spiked in 5 mL peripheral blood (LOD = 1 CTC/5mL peripheral blood) can be retrieved by the platform, which indicated its high sensitivity for CTC detection.

To better characterize platform precision, the coefficient of variation (% CV) of recovery rate was evaluated across 3 separate days (*n* = 3) by two operators (*n* = 2) using paired blood samples. The results of these intra‐ and inter‐assays showed the high repeatability of the platform; the calculated % CVs were 12.4% for Operator A and 11.1% for Operator B (*P* > 0.05, Fig. 3C). Blood samples from HDs were added with high levels of endogenous interfering reagents to evaluate the anti‐interference capability of the platform. The differences in average recovery rates between groups with and without interfering reagents were less than 10% (*P* > 0.05, Fig. 3D). No false‐positive tumor cells were found in un‐spiked healthy blood in our preclinical experiment, thereby showing assay specificity.

### 3.2 Comparison of different methods for CTC detection

Because positive enrichment is one of the most common approaches used in CTC detection, we tested the performance of the ChimeraX^®^‐i120 platform and CellSearch system in CTC detection. First, relative EpCAM expression levels of four cancer cell lines (SkBr3, HT‐29, HepG2, and T24) were determined using immunofluorescence labeling (Fig. 3E,F). To allow for a direct comparison, each of the four cell lines was spiked separately in blood drawn in parallel from HDs and enriched using the CellSearch system or ChimeraX^®^‐i120 platform. The results indicated that the CellSearch system yielded a recovery rate similar to the ChimeraX^®^‐i120 platform only for the HT‐29 cells (72.3% *vs*. 55.7%, *P* = 0.124), a cell line with high EpCAM expression. The recovery rates of cell lines with low or intermediate EpCAM expression were significantly lower than those of the ChimeraX^®^‐i120 platform (T24, 10.7% *vs*. 60.7%, *P* < 0.001; SkBr3, 44.3% vs. 78.0%, *P* = 0.003; HepG2, 39.3% vs. 59.7%, *P* = 0.006). However, the ChimeraX^®^‐i120 platform generated a similar recovery rate of 67.7% (range, 59.7–78.0%) across the four cell lines, regardless of their EpCAM expression level (Fig. 3G).

A small cohort containing twenty‐three cancer patients (HCC, *n* = 14; ICC, *n *= 5; CRC, *n* = 4) were then enrolled in the comparison assay (Table S1). Using the CellSearch system, 10 of 23 (43.5%) patients had detectable CTCs. A blinded comparison of paired samples revealed that the ChimeraX^®^‐i120 platform had more captured CTCs (mean, 2.09 ± 1.91 *vs*. 0.83 ± 1.19, respectively, *P* = 0.004) and higher positive rates (69.5% *vs*. 43.5%, respectively, *P* = 0.031, Fig. 3H). A preliminary comparative assessment was made in this study, and these results indicated that a negative CTC sorting strategy may reduce the cell loss during CTC enrichment.

### 3.3 The throughput of blood samples processing and machine learning‐based identification of CTC

To fulfill the clinical demands of throughput and efficiency for CTC detection, we built a streamlined workflow and optimized many parameters in blood sample preparation, CTC isolation, immunofluorescence labeling, and CTC identification from images. It takes about four hours to process up to six blood samples simultaneously for our approach.

The integrated high‐content fluorescent screening platform enabled per cell high‐definition image analyses after CTC enrichment by the ChimeraX^®^‐i120 platform. Original screened fluorescent images of the enriched cells were analyzed via a customized image analysis pipeline (Fig. 2B), during which the images were automatically preprocessed for further analyses. Because manual CTC recognition from numerous images is time‐consuming and laborious, we additionally developed a method by machine learning‐based CTC identification to find and distinguish tumor from nontumor cells easily. The detailed principle for the development of the machine learning‐based classifiers is presented in Fig. S1a. All the five classifiers achieved high performance according to the ROC curve analyses (area under the curve (AUC) > 0.99). Among these classifiers, Classifier XGB resulted in the highest recall value (99.3%, Fig. S1b). With the help of this method, the standard CTC test report can be generated after a simple confirmation by the pathologist.

### 3.4 Validation of CTC detection in blood samples from cancer patients

**Fig. 4:**
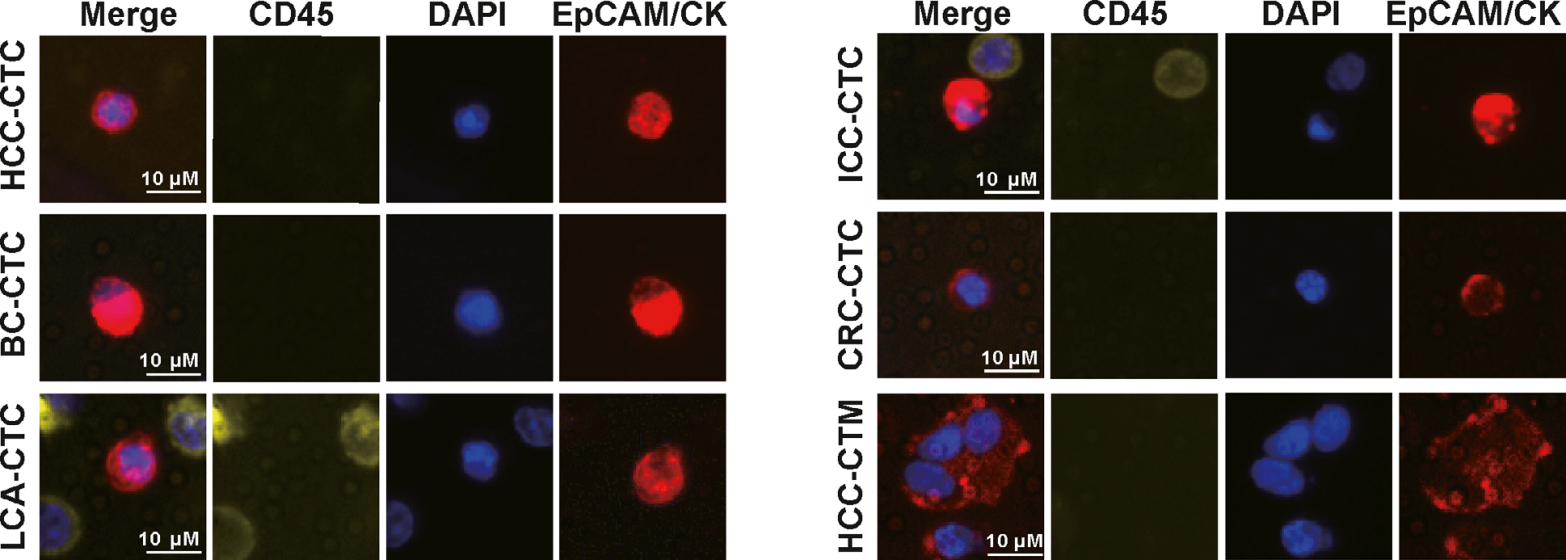
Representative images of detected CTCs and circulating tumor microemboli. Scale bar, 10 μm.

**Fig. 5:**
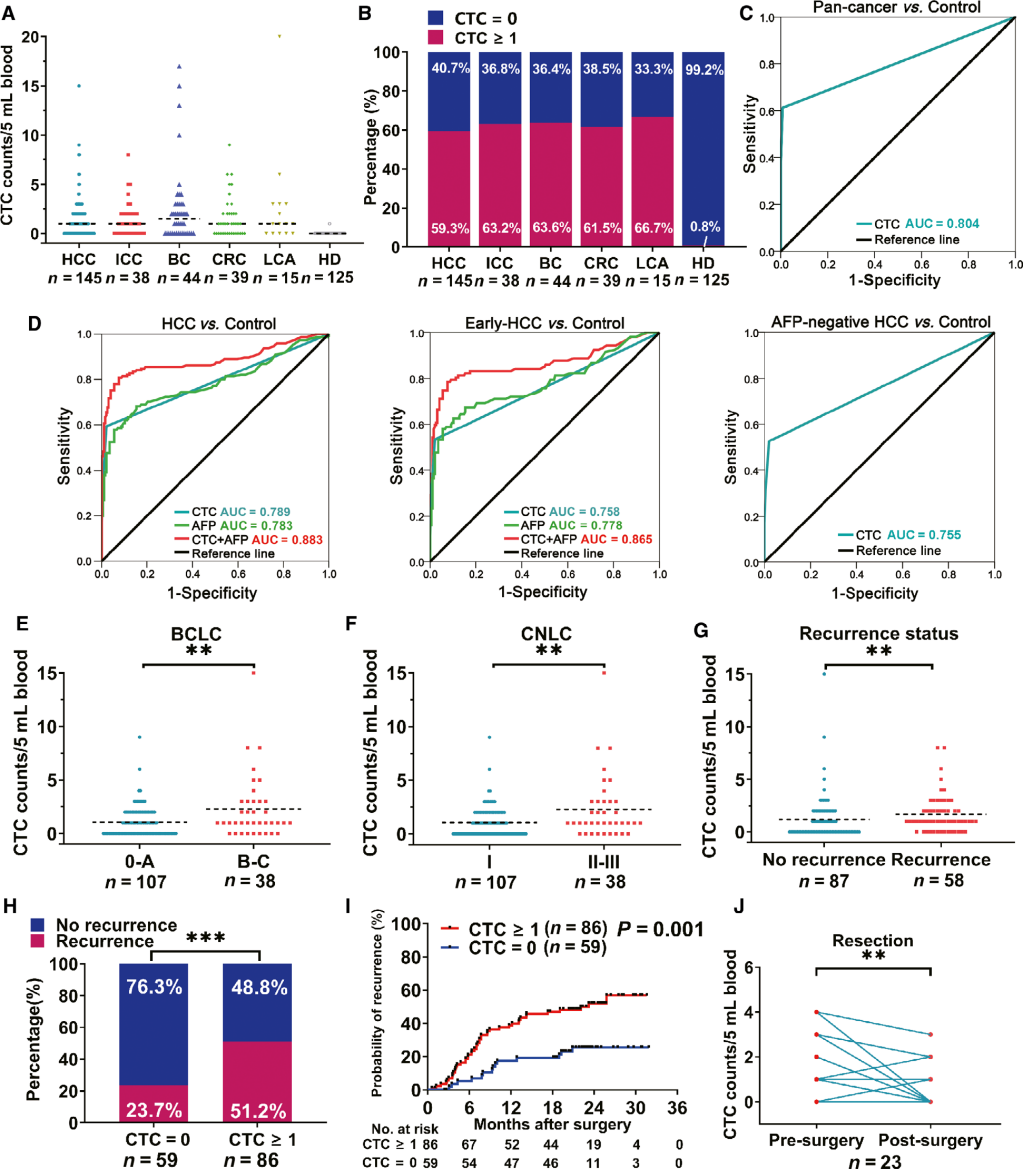
Clinical evaluation of ChimeraX^®^‐i120 platform in CTC detection. (A) CTC count of patients with different type of cancers (*n* = 281) and healthy donors (*n* = 125). (B) CTC‐positive rate (CTC ≥ 1) of patients with different types of cancer (*n* = 281) and healthy donors (*n* = 125). (C) ROC analysis of CTC detection in pan‐cancer diagnosis (pan‐cancer *vs*. HD). (D) ROC of CTC detection in HCC diagnosis. From left to right: HCC *vs*. CHB/LC + BHL+HD, early HCC *vs*. CHB/LC + BHL+HD, AFP‐negative HCC *vs*. CHB/LC + BHL+HD. (E) CTC count of HCC patients with BCLC 0‐A and B‐C stage (*n* = 145, Mann–Whitney test). (F) CTC count of HCC patients with CNLC stage Ⅰ and stages Ⅱ‐Ⅲ(*n* = 145, Mann–Whitney test). (G) CTC count of HCC patients with (*n* = 58) or without recurrence (*n* = 87, Mann–Whitney test). (H) Recurrence ratio of HCC patients with CTC ≥ 1 (*n* = 86) or CTC = 0 (*n* = 59) during the follow‐up period (chi‐squared test). (I) The Kaplan–Meier analysis of rates of recurrence in HCC patients (*n* = 145) stratified by CTC status, differences between groups were assessed using log‐rank tests. (J) CTC count of HCC patients before and after curative resection (paired Wilcoxon signed‐rank test). Significance is indicated by ^**^ *P* < 0.010 and ^***^ *P* < 0.001. Scale bar, 10 μm.

The performance of ChimeraX^®^‐i120 platform for CTC detection for multiple indications was validated. A total of 406 participants including 281 patients with five cancer types (HCC, ICC, CRC, BC, and LCA) and 125 healthy individuals were recruited into the test cohort (Table S2). Representative images of CTCs and circulating tumor microemboli (CTM) from a patient with HCC are presented in Fig. 4. The results showed that the platform could detect CTCs from a broad spectrum of malignancies with similar positive CTC rates (Fig. 5A, B). CTCs were detected in 59.3% (86/145; mean, 1.37 ± 2.01) of HCC patients, 63.2% (24/38; mean, 1.42 ± 1.75) of ICC patients, 63.6% (28/44; mean, 2.52 ± 3.93) of BC patients, 61.5% (24/39; mean, 1.62 ± 2.11) of CRC patients, and 66.7% (10/15; mean, 2.73 ± 5.06) of LCA patients; only one CTC was detected in the blood of a healthy individual (1/125, 0.8%). ROC curves for the entire cancer patient cohort and the controls (i.e., HDs) are presented in Fig. 5C. The AUC for cancer diagnosis was 0.804 (95% confidence interval (CI) 0.76–0.84; optimal sensitivity of 61.2% and specificity of 99.2%).

### 3.5 Clinical feasibility of CTC detection for diagnosis, prognosis prediction, and treatment response surveillance in patients with HCC

The clinical feasibility of the platform was further investigated in a combined cohort consisted of the patients with HCC (*n* = 145), and the patients with CHB/LC (*n* = 31), and BHL (*n* = 40), and HDs (*n* = 125). The CTC‐positive rates were 6.5% (2/31, mean, 0.10 ± 0.40) in the CHB/LC group and 2.5% (1/40, mean, 0.03 ± 0.16) in the BHL group (Fig. S2). The calculated AUC for CTCs in HCC diagnosis was 0.789 (95% CI = 0.74–0.84, *P* < 0.001, Fig. 5D). When CTCs were combined with serum AFP level, the diagnostic value improved and the AUC was 0.883 (95% CI = 0.84–0.93, *P* < 0.001; optimal sensitivity of 80.7% and specificity of 92.3%). More importantly, CTCs were identified in 53.3% (57/107) of early‐stage HCC patients and 52.6% (40/76) of AFP‐negative (AFP ≤ 20 ng·mL^−1^) HCC patients. These results suggested that CTC analysis may help identify early‐stage HCC (AUC = 0.758) and AFP‐negative HCC (AUC = 0.755, Fig. 5D).

The clinical characteristics of the HCC patients who underwent curative resection are presented in Table 2. The patients were divided into two groups based on preoperative CTC status (CTC = 0 and CTC ≥ 1). We found that the patients with CTCs ≥ 1 were more likely to have tumors with advanced characteristics including larger size (*P* = 0.007), vascular invasion (*P* = 0.001), advanced stage (BCLC, *P* = 0.013; CNLC, *P* = 0.013), and multiple tumors (*P* = 0.094). Advanced HCC was also associated with higher CTC counts (*P* = 0.007 for BCLC, Fig. 5E; *P* = 0.007 for CNLC, Fig. 5F).

**Table 2:** Baseline characteristics of HCC patients undergoing curative resection

Variable		CTC = 0	CTC ≥ 1	*P*
		(*n* = 59)	(*n* = 86)	
Gender	Male	50 (84.7%)	76 (88.4%)	0.525
	Female	9 (15.3%)	10 (11.6%)	
Age (years)	≤50	9 (15.3%)	20 (23.3%)	0.237
	>50	50 (84.7%)	66 (76.7%)	
Tumor number	Single	49 (83.1%)	61 (70.9%)	0.094
	Multiple	10 (16.9%)	25 (29.1%)	
Tumor diameter (cm)	≤5	47 (79.7%)	50 (58.1%)	**0.007**
	>5	12 (20.3%)	36 (41.9%)	
Tumor capsule	Complete	37 (62.7%)	46 (53.5%)	0.270
	None	22 (37.3%)	40 (46.5%)	
Vascular invasion	No	43 (72.9%)	38 (44.2%)	**0.001**
	Yes	16 (27.1%)	48 (55.8%)	
Edmondson stage	Ⅰ‐Ⅱ	30 (50.8%)	36 (41.9%)	0.286
	Ⅲ‐Ⅳ	29 (49.2%)	50 (58.1%)	
Liver cirrhosis	No	28 (47.5%)	46 (53.5%)	0.475
	Yes	31 (52.5%)	40 (46.5%)	
HBsAg	Negative	14 (23.7%)	29 (33.7%)	0.196
	Positive	45 (76.3%)	57 (66.3%)	
HBV DNA (IU/mL)	≤10	37 (62.7%)	57 (66.3%)	0.659
	>10	22 (37.3%)	29 (33.7%)	
AFP (ng/mL)	≤400	48 (81.4%)	64 (74.4%)	0.328
	>400	11 (18.6%)	22 (25.6%)	
ALB (g/L)	≤34	1 (1.7%)	2 (2.3%)	1.000^a^
	>35	58 (98.3%)	84 (97.7%)	
ALT (U/L)	≤50	49 (83.1%)	67 (77.9%)	0.447
	>50	10 (16.9%)	19 (22.1%)	
Child‐Pugh	A	59 (100.0%)	86 (100.0%)	1.000
Class	B	0 (0.0%)	0 (0.0%)	
BCLC stage	0‐A	50 (84.7%)	57 (66.3%)	**0.013**
	B‐C	9 (15.3%)	29 (33.7%)	
CNLC stage	Ⅰ	50 (84.7%)	57 (66.3%)	**0.013**
	Ⅱ‐Ⅲ	9 (15.3%)	29 (33.7%)	

AFP, alpha‐fetoprotein; ALB, albumin; ALT, alanine aminotransferase; BCLC, Barcelona Clinic Liver Cancer Staging System; CNLC, Chinese Liver Cancer Staging System; HBsAg, hepatitis B surface antigen.

^a^ Continuous correction.

Bold values indicate *P*‐value < 0.05.

The prognostic value of CTC counts was then investigated. With a median follow‐up time of 20.5 months, 40.0% (58/145) of the patients experienced tumor recurrence. We found that CTC counts were significantly higher in patients with tumor recurrence as compared with those without recurrence (mean, 1.67 ± 1.79 *vs*. 1.18 ± 2.13, median, 1.00 *vs*. 0, respectively; *P* = 0.006, Fig. 5G); 44 of 86 (51.2%) patients with preoperative CTC ≥ 1 developed HCC recurrence (Fig. 5H). The Kaplan–Meier analysis revealed that CTC‐positive patients had higher cumulative recurrence rates (56.8% *vs*. 25.5%, respectively, *P* = 0.001) and shorter median TTR (23.2 months *vs*. not reached, *P* = 0.001) than those without detectable CTCs (Fig. 5I). Moreover, twenty‐three patients accepted an additional CTC tests at 1 month after surgery, and postoperative CTC burdens of most patients decreased dramatically (positive rate: 21.7% *vs*. 69.6%, respectively; mean: 0.39 ± 0.84 *vs*. 1.30 ± 1.26, median, 0 *vs*. 1.00, respectively; *P* = 0.002, Fig. 5J).

### 3.6 Downstream analyses of single tumor cells after enrichment

The ChimeraX^®^‐i120 platform had four changeable channels that allowed for multiple protein biomarkers CTC labeling. In addition to CTC enumeration, the CTCs enriched by our ChimeraX^®^‐i120 platform were compatible with downstream cellular morphology evaluation (e.g., Fig. 3E, F), multi‐biomarker expression measurements, and single‐cell molecular analyses.

**Fig. 6:**
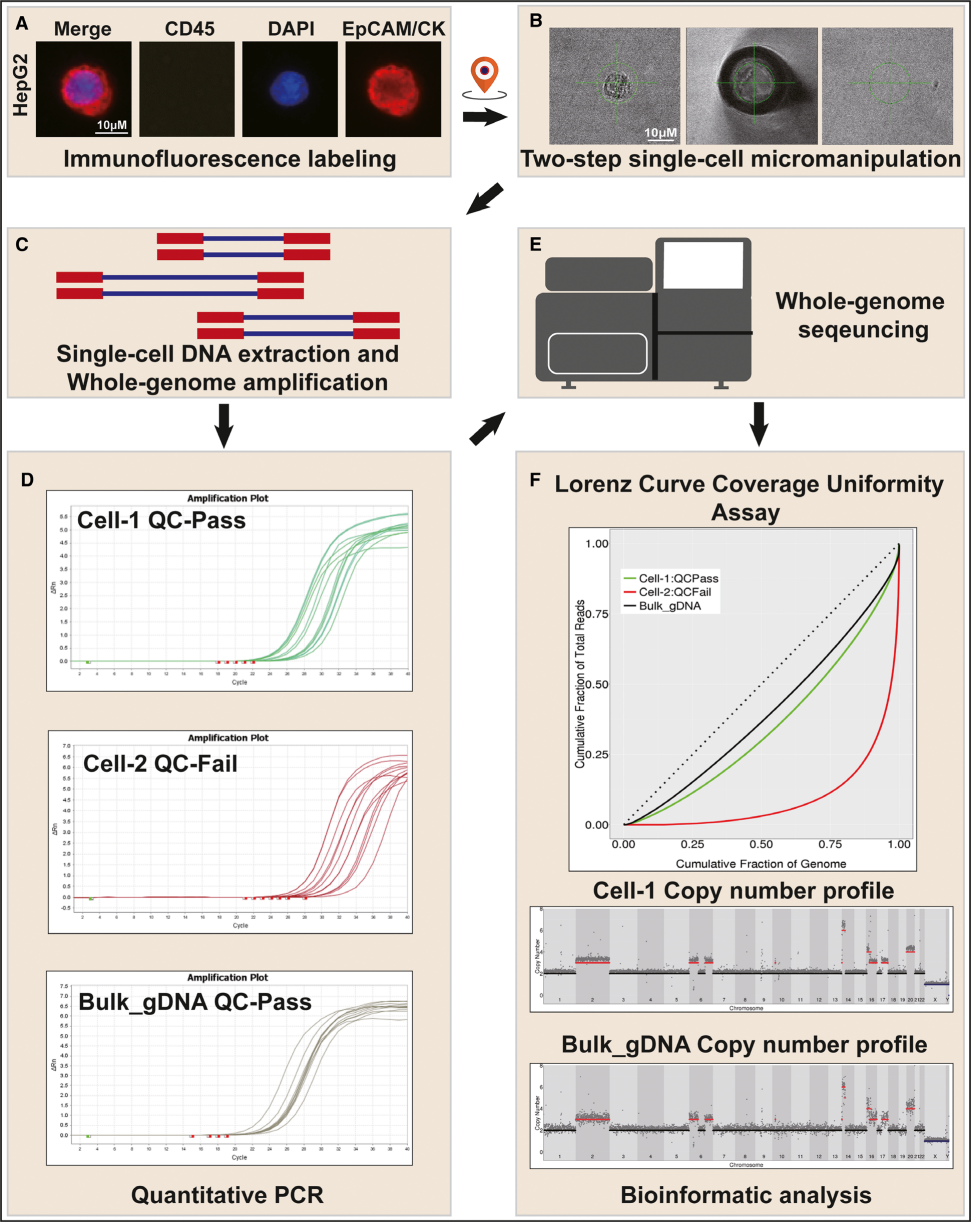
ChimeraX^®^‐i120 platform CTC single‐cell genomic analysis pipeline. (A) Immunofluorescence labeling and screening of CTC, coordinates of cells of interest can be generated. (B) Two‐step single‐cell micromanipulation by CellCelector platform. (C) Schematic diagram of single‐cell library preparation. (D) Typical amplification curve of QC‐Passed single HepG2 cell (Cell‐1) and pooled HepG2 cells, QC‐Failed single HepG2 cell (Cell‐2) in quantitative PCR step for quality control. (E) Schematic diagram of whole‐genome sequencing. (F) Bioinformatic analysis of sequencing data. Representative images showed the results of Lorenz curve coverage uniformity assay (top), copy number profile of single HepG2 cell (Cell‐1, mid), and copy number profile of pooled HepG2 cells (Bulk_gDNA, bottom). Scale bar, 10 μm.

Here, we used spiked HepG2 cells to present the working principle of our optimized single CTC CNA analysis via low‐pass single‐cell whole‐genome sequencing (WGS) [30] (Fig. 6). After CTC enrichment, fluorescence‐labeled cells were collected by a CellCelector platform. The prior image analysis pipeline could output coordinates of cells of interest, which helps the operator of the CellCelector find where cells locate in the well easily. This approach allowed for accurate CTC positioning and minimally invasive single‐cell collection, retained the integrity of the isolated cell. Once identified, each target cell was automatically isolated from the bulk population containing cells, platelets, and debris, following a customized two‐step isolation protocol: The robot arm was programmed to first aspirate the CTCs from the sample region and deposit them into a new well containing PBS. Thus, the sample was diluted and allowed for a second, more purified single‐cell CTC collection (Fig. 6A,B and Video S3). The total time and capture efficiency of this step were approximately 10 min and > 85%, respectively. Then, rigorous quality control criteria were followed at every step to ensure the followed single‐cell sequencing accuracy and efficiency (Fig. 6C–F). Copy number profiles of single HepG2 cell and pooled HepG2 cells were plotted (Fig. 6F), and most CNAs were matched (*r* = 0.954).

**Fig. 7:**
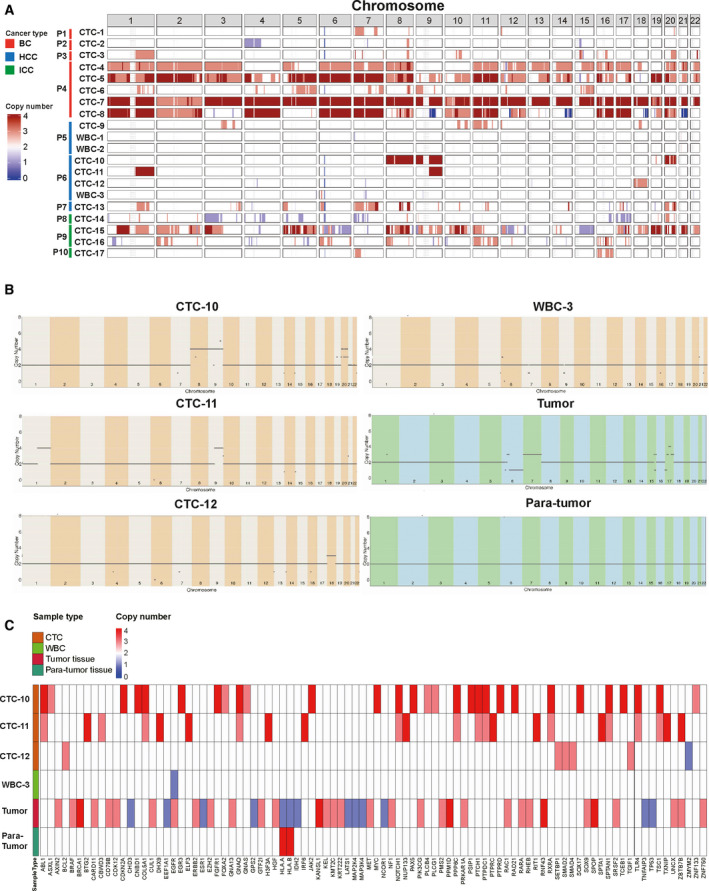
Genomic analysis of single CTCs and tissues from cancer patients. (A) CNA pattern of 17 CTCs and 3 WBCs. (B) CNA profile of CTCs, WBC, and paired tumor, para‐tumor tissue of patient P6. (C) CNA analysis to explore chromosomal region that may contain potentially actionable or cancer driver genomic alterations of patient P6.

To test the feasibility of single CTC CNA analyses in the clinical setting, a total of 17 CTCs and 3 white blood cells (WBCs) were picked from 10 patients with cancer (3 HCC, 3 ICC, and 4 BC) and subjected to CNA analysis. These CTCs exhibited heterogeneous chromosomal CNA distribution patterns. Distinctly different CNA patterns were observed between CTCs from two patients (P5 and P6) with HCC and their matched normal WBCs (Fig. 7A).

One patient with HCC was further selected to investigate the relationship between primary tumor tissue and CTCs. The same sequencing assay as that used for CTCs was performed for resected tumor and para‐tumor tissue. We found that compared with the primary tumor tissue, some CTCs had a different CNA pattern (Fig. 7B). Multiple HCC‐associated oncogenes (e.g., Notch1, ABL1, JAK2, FGFR1, and MYC; Fig. 7C) were exclusively identified in the CTCs at regions with high copy number [31, 32, 33, 34]. These specific genomic alterations may be responsible for CTC survival or initiation of tumor metastasis.

## 4 Discussion

Applying comprehensive CTC analyses to guide cancer diagnostics and genome‐informed therapeutics in the clinical setting is promising but remains challenging because of the requirements for highly reliable methods. In the study, we built and optimized the ChimeraX^®^‐i120 platform for CTC detection. Critical analytical assessment of the platform on CTC enrichment was conducted by evaluating a series of assay performance characteristics. Its compatibilities for CTC enumeration and downstream single CTC profiling for multiple cancers were validated. Our image and molecular analysis workflow facilitate a simultaneous evaluation of the target protein expression, morphology characteristics, and genomic alterations of a single CTC. Thus, the ChimeraX^®^‐i120 platform and the established workflow represent a reliable solution for comprehensive CTC analyses in the clinical setting.

Numerous systems have been designed to enumerate CTCs in the last 10 years. However, many are tumor antigen‐dependent or require multiple batch processes in laboratories, or both. These characteristics may narrow their potential applications due to great cell loss and time costs [14, 35]. Because CTC subpopulations exhibit great heterogeneity, it is rational to combine negative enrichment and biomarker identification to minimize cell loss and decrease the false‐positive CTC detection rates [16]. Following this strategy, our platform used negative enrichment and the canonical tumor marker EpCAM and cytokeratin, among other markers, which have been regarded as clinical standards in CTC labeling [14, 36]. Many recently developed CTC platforms also rely on the epithelial marker for CTC labeling, since some epithelial‐to‐mesenchymal markers (i.e., vimentin) are also expressed on the surrounding leukocytes [15]. Some researchers also indicated that epithelial features were required for CTCs to seed distant metastases [37]. The preclinical results from the spike‐in experiments demonstrated that the ChimeraX^®^‐i120 platform achieved an unbiased enrichment of CTC subpopulations with insufficient expression of EpCAM. This result was less likely with an EpCAM microbead‐based magnetic sorting method (e.g., CellSearch). Thus, the negative selection mode of the platform may better provide a comprehensive and unbiased view of tumor cells in the bloodstream of cancer patients.

Standardized CTC isolation and enumeration protocols are necessary for direct CTC comparisons between multiple measurements for cancer patients (e.g., therapeutic response monitoring, recurrence surveillance) [38]. During platform design, reproducibility and automation were incorporated to ensure the robustness of CTC enrichment. The combination of the automatic pipetting unit and CCD camera for interface recognition enables stable and precise liquid handling after the centrifugation, minimizes the possibility of cell loss and contamination of blood cells, while the manual collection of the CTC enriched layer is can be highly variable. Beginning with spiking experiments, a series of analytical validation tests demonstrated that the platform had high accuracy, reproducibility (intra‐assay and interassay), specificity, and anti‐interference capability for CTC detection. Meanwhile, manual identification of CTCs from numerous images remains user‐dependent and time‐consuming, which is a common point of criticism for traditional CTC enrichment platforms [27, 39]. Defining standardized recognition criteria for CTCs is also essential for clinical applications. To address this issue, we showed a method for CTC identification powered by machine learning. The proprietary method utilized multiple graphic features (such as morphologic parameters, marker expressions) to recognize and pinpoint CTCs, which enabled an automatic or operator‐assisted identification of desired cells and minimized the possibility of selecting inappropriate objects such as debris or noise signal. The outstanding results of these evaluation parameters (e.g., AUC, recall, precision, F1 score) of the machine learning‐based classifiers demonstrated high concordance between this method and manual identification of CTCs. Also, a recent study reported their machine learning‐based CTC recognition protocols with more than 90% sensitivity and specificity [39]. We believe this method could effectively reduce the time required for manual image screening, reduce artificial errors, and potentialize standardized CTC recognition in clinical practice.

CTC enumeration is a widely used noninvasive marker for cancer diagnosis [40]. In our study, the ChimeraX^®^‐i120 platform yielded a similar between‐cancer CTC‐positive rate of 60%; false‐positive CTCs were rarely found in healthy individuals (1/125, 0.8%), suggesting the diagnostic use of CTCs. The CTCs detected also exhibited moderate diagnostic potential for differentiating patients with HCC from healthy individuals and patients with CHB/LC and BHL, with enhanced diagnostic efficiency if combined with a traditional serological biomarker (i.e., AFP). These results supported the use of the platform as a complementary tool to assist in the screening and early diagnosis of cancers. However, considering the limited cohort size of the present study, further well‐designed, multicenter, large population study is still warranted to validate the diagnostic value of CTC.

Other than the utility for cancer diagnosis, CTC detection could provide valuable information for real‐time cancer status evaluation and prognosis prediction, these applications were also demonstrated in this study [41]. Globally, HCC is one of the most frequently diagnosed malignancies [1, 22]. Patients with HCC who undergo curative resection still suffer from a high incidence of tumor recurrence [22]. We previously reported that preoperative EpCAM^+^ CTC numbers could predict prognosis in patients with HCC [16, 42]. In this study, the prognostic significance of CTCs detected using the ChimeraX^®^‐i120 platform was also confirmed. The decrease in CTC load was also observed soon after resection, which is usually associated with a decreased probability of recurrence [19].

Downstream analysis of individual cancer cells provides a new approach for exploring tumor biological phenotypes [43]. By combining the ChimeraX^®^‐i120 platform and the integrated workflow, we could obtain reliable and qualified single CTCs for many downstream molecular analyses (e.g., PCR, next‐generation sequencing).

In this proof‐of‐concept study, diverse CNA patterns of single CTCs were identified in 10 cancer patients by low‐pass sequencing. Such genomic discrepancies in CTCs might be attributed to intra‐ and intertumor heterogeneity, which would be too minor to be depicted using traditional pathologic methods [44, 45]. In patient P6, CNA analysis revealed distinct patterns between CTCs and paired WBCs or para‐tumoral tissue. This result also demonstrated the potential of CTC sequencing as a noninvasive method in differentiating benign and malignant diseases in the clinic (Fig. 7A). Another key finding from this patient with HCC was that some significant genomic alterations were exclusively found in the CTCs, but not in the matched tissues (Fig. 7C). Indeed, some reports showed that the dissemination of CTCs is likely not a random, but is a convergent evolution process during tumor development. Sophisticated genomic rearrangements are required before primary tumor cells turn to CTCs [44, 45, 46]. Thus, we hypothesized that these CTCs analyzed were subclonal origin from the primary tumor, and these significant genomic alterations conferred them metastatic potential and survival advantages [8, 9, 47]. Therefore, with such heterogeneity unveiled from single‐cell sequencing analysis, we envisioned that our approach for single CTC genomic profiling will not only be useful for studying tumor evolution and dissemination but will also be a powerful tool to enable the personalized therapeutics of cancer.

This study reported an integrated workflow for robust CTC enrichment, identification, and downstream single‐cell analysis. However, it did have some limitations. We used a relatively small cohort size with a short follow‐up time for validation, and the data were from a single medical center. The clinical significance of our protocols for CTC detection was only evaluated in patients with HCC. How well the platform and the established workflow perform across other malignant tumors need to be further evaluated in large‐scale, prospective, and well‐designed studies.

## 5 Conclusions

Altogether, the ChimeraX^®^‐i120 platform and the standardized workflow enable precise, highly automated single CTC enumeration and molecular characterization. It represents a readily accessible and clinically feasible approach to make CTC detection an essential tool to provide real‐time clinical information.

## Conflicts of interest

Wei‐Xiang Jin, Hai‐Xiang Peng, Li‐Meng Chen, and Kai Huang are employees of Shanghai Epione Medlab. The remaining authors have no conflicts of interest to declare.

## Author contributions

PXW and YFS conceptualized the data, provided software, involved in formal analysis, designed methodology, visualized the data, wrote the original manuscript, and prepared the draft; WXJ involved in formal analysis, provided software, designed methodology, and wrote the original draft; JWC, YX, KQZ, SYW, BH, ZFZ, and WG investigated the data, administered the project, and validated the data; HXP, LMC, and KH investigated the data, involved in formal analysis, administered the project, and provided software; YC conceptualized the data and supervised the data; JZ conceptualized the data, designed methodology, supervised the data, and acquired funding; and JF and XRY provided resources, conceptualized the data, designed methodology, acquired funding, supervised the data, wrote, reviewed, and edited the manuscript. All authors have read and agreed to the published version of the manuscript.

## Supporting information

**Fig S1.** Supervised machine learning classifier training and evaluation.

**Fig S2.** Results of CTC detection by ChimeraX^®^‐i120 platform for HCC diagnosis.

**Table S1.** Comparison of ChimeraX^®^‐i120 platform with CellSearch system in paired blood samples from cancer patients.

**Table S2.** Distribution and CTC count of cancer patients.

**Video S1.** Automatic CTC detection by ChimeraX^®^‐i120 platform.

**Video S2.** Machine learning based CTC identification.

**Video S3.** Single CTC micro‐manipulation.

## Data Availability

The data that support the findings of this study are available from the corresponding author upon reasonable request.
