# Supplementary figures and images for: Circulating tumor cell detection and single‐cell analysis using an integrated workflow based on ChimeraX®‐i120 Platform: A prospective study
Source: Mol Oncol. 2020 Dec 25;15(9):2345–62. doi: 10.1002/1878-0261.12876 (PMC8410565; doi:10.1002/1878-0261.12876)

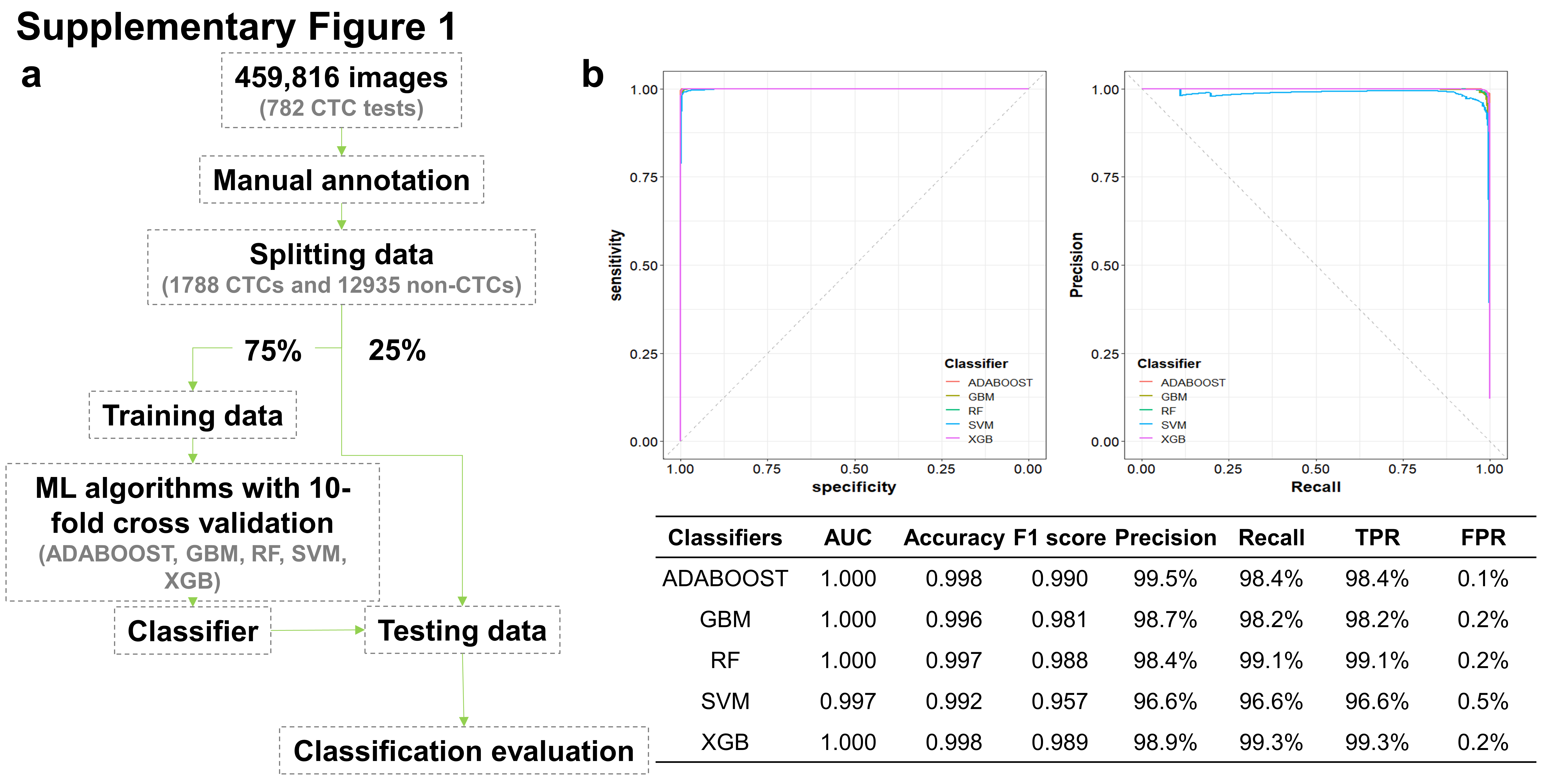

Supplement: Supplementary file 1 — Fig S1. Supervised machine learning classifier training and evaluation. [file MOL2-15-2345-s002.tif]

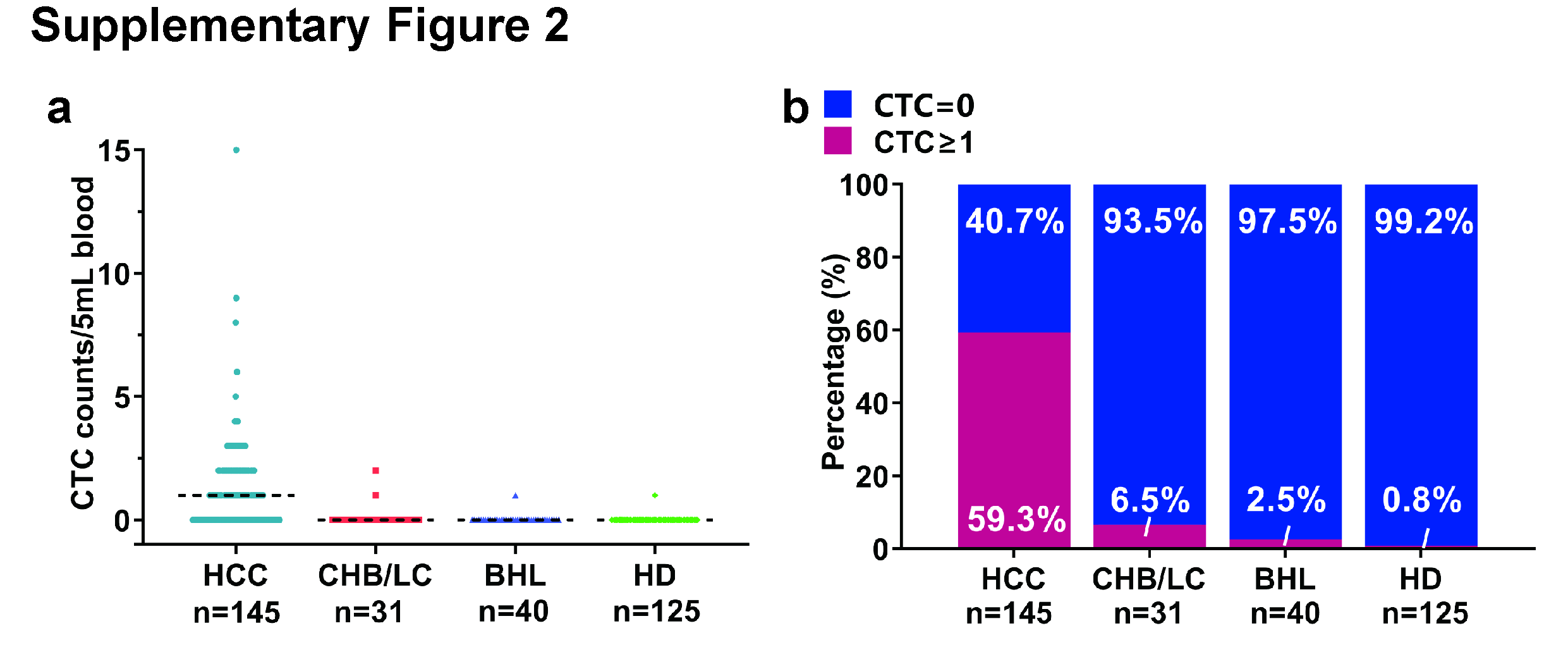

Supplement: Supplementary file 2 — Fig S2. Results of CTC detection by ChimeraX®‐i120 platform for HCC diagnosis. [file MOL2-15-2345-s004.tif]
