# Supplementary material for: Circulating tumor cell detection and single‐cell analysis using an integrated workflow based on ChimeraX®‐i120 Platform: A prospective study
Source: Mol Oncol. 2020 Dec 25;15(9):2345–62. doi: 10.1002/1878-0261.12876 (PMC8410565; doi:10.1002/1878-0261.12876)
Supplement: Supplementary file 3 — Table S1. Comparison of ChimeraX®‐i120 platform with CellSearch system in paired blood samples from cancer patients. [file MOL2-15-2345-s005.docx]

**Supplementary Table 1. Comparison of ChimeraX^®^-i120 platform with CellSearch system in paired blood samples from cancer patients.**

| **Patient ID** | **Cancer type** | **Age** | **Gender** | **AJCC stage** | **CTC count/5mL**  **(ChimeraX^®^-i120)** | **CTC count/7.5mL**  **(CellSearch)** |
| --- | --- | --- | --- | --- | --- | --- |
| P1 | HCC | 58 | Male | I | 0 | 0 |
| P2 | ICC | 69 | Male | II | 0 | 0 |
| P3 | HCC | 63 | Male | II | 1 | 2 |
| P4 | HCC | 59 | Male | I | 4 | 1 |
| P5 | HCC | 52 | Male | I | 0 | 0 |
| P6 | CRC | 63 | Male | II | 1 | 2 |
| P7 | HCC | 73 | Female | III | 5 | 3 |
| P8 | ICC | 52 | Male | III | 1 | 3 |
| P9 | ICC | 60 | Female | III | 4 | 4 |
| P10 | HCC | 61 | Male | III | 5 | 1 |
| P11 | HCC | 53 | Male | I | 2 | 1 |
| P12 | HCC | 55 | Male | II | 2 | 0 |
| P13 | CRC | 48 | Male | I | 0 | 0 |
| P14 | HCC | 61 | Male | I | 0 | 0 |
| P15 | ICC | 53 | Male | I | 0 | 0 |
| P16 | ICC | 71 | Male | II | 3 | 0 |
| P17 | HCC | 38 | Female | I | 0 | 0 |
| P18 | CRC | 73 | Female | II | 5 | 1 |
| P19 | HCC | 50 | Female | I | 3 | 0 |
| P20 | HCC | 51 | Male | III | 3 | 1 |
| P21 | HCC | 47 | Male | I | 1 | 0 |
| P22 | HCC | 61 | Male | II | 3 | 0 |
| P23 | CRC | 79 | Male | III | 5 | 0 |

**Abbreviations:** AJCC, American Joint Committee on Cancer; CTC, circulating tumor cell; HCC, hepatocellular carcinoma; ICC, intrahepatic cholangiocarcinoma; CRC, colorectal cancer.
