# Supplementary material for: Circulating tumor cell detection and single‐cell analysis using an integrated workflow based on ChimeraX®‐i120 Platform: A prospective study
Source: Mol Oncol. 2020 Dec 25;15(9):2345–62. doi: 10.1002/1878-0261.12876 (PMC8410565; doi:10.1002/1878-0261.12876)
Supplement: Supplementary file 4 — Table S2. Distribution and CTC count of cancer patients. [file MOL2-15-2345-s007.docx]

| **Tumor Type** | **AJCC**  **Stage** | **Patients (n, %)** | **Mean**  **CTC** | **SD** | **Range** | **Median** |
| --- | --- | --- | --- | --- | --- | --- |
| Breast cancer | Total | 44 | 2.52 | 3.92 | 0-17.00 | 1.50 |
|  | I | 14 (31.80%) | 1.85 | 3.97 | 0-15.00 | 0.00 |
|  | II | 21 (47.70%) | 2.85 | 4.68 | 0-17.00 | 1.00 |
|  | III | 6 (13.60%) | 2.83 | 1.16 | 2-3.00 | 2.50 |
|  | IV | 3 (6.80%) | 2.66 | 1.15 | 2-4.00 | 2.00 |
| Colorectal cancer | Total | 39 | 1.61 | 2.11 | 0-9.00 | 1.00 |
|  | I | 12 (30.80%) | 0.58 | 0.66 | 0-2.00 | 0.50 |
|  | II | 13 (33.30%) | 1.23 | 1.48 | 0-5.00 | 1.00 |
|  | III | 10 (25.60%) | 2.30 | 2.16 | 0-6.00 | 2.00 |
|  | IV | 4 (10.30%) | 4.25 | 4.03 | 0-9.00 | 4.00 |
| Hepatocellular carcinoma | Total | 145 | 1.37 | 2.01 | 0-15.00 | 1.00 |
|  | I | 99 (68.30%) | 1.07 | 1.40 | 0-9.00 | 1.00 |
|  | II | 23 (15.90%) | 1.91 | 3.38 | 0-15.00 | 1.00 |
|  | III | 23 (15.90%) | 2.17 | 2.18 | 0-8.00 | 1.00 |
|  | IV | 0 (0.00%) | N/A | N/A | N/A | N/A |
| Intrahepatic cholangiocarcinoma | Total | 38 | 1.42 | 1.75 | 0-8.00 | 1.00 |
|  | I | 11 (28.90%) | 0.81 | 1.07 | 0-3.00 | 0.00 |
|  | II | 12 (31.60%) | 1.00 | 1.12 | 0-3.00 | 0.50 |
|  | III | 12 (31.60%) | 1.50 | 1.50 | 0-5.00 | 1.00 |
|  | IV | 3 (7.90%) | 5.00 | 3.00 | 2-8.00 | 5.00 |
| Lung cancer | Total | 15 | 2.73 | 5.06 | 0-20.00 | 1.00 |
|  | I | 4 (26.70%) | 0.50 | 0.57 | 0-1.00 | 0.50 |
|  | II | 4 (26.70%) | 1.00 | 1.41 | 0-3.00 | 0.50 |
|  | III | 5 (33.30%) | 2.40 | 2.30 | 0-6.00 | 2.00 |
|  | IV | 2 (13.30%) | 11.5 | 12.0 | 3-20.00 | 11.5 |

**Supplementary Table 2. Distribution and CTC count of cancer patients.**

**Abbreviations:** CTC, circulating tumor cells; AJCC, American Joint Committee on Cancer; SD, standard deviation.
